# The Key Role of MicroRNAs in Self-Renewal and Differentiation of Embryonic Stem Cells

**DOI:** 10.3390/ijms21176285

**Published:** 2020-08-31

**Authors:** Giuseppina Divisato, Fabiana Passaro, Tommaso Russo, Silvia Parisi

**Affiliations:** Department of Molecular Medicine and Medical Biotechnology, University of Naples Federico II, Via S. Pansini 5, 80131 Naples, Italy; giuseppina.divisato@unina.it (G.D.); fabiana.passaro@unina.it (F.P.); tommaso.russo@unina.it (T.R.)

**Keywords:** microRNAs, embryonic stem cells, induced pluripotent stem cells, naïve pluripotency, epiblast-stem cells, cell reprogramming, differentiation

## Abstract

Naïve pluripotent embryonic stem cells (ESCs) and epiblast stem cells (EpiSCs) represent distinctive developmental stages, mimicking the pre- and the post-implantation events during the embryo development, respectively. The complex molecular mechanisms governing the transition from ESCs into EpiSCs are orchestrated by fluctuating levels of pluripotency transcription factors (*Nanog*, *Oct4*, etc.) and wide-ranging remodeling of the epigenetic landscape. Recent studies highlighted the pivotal role of microRNAs (miRNAs) in balancing the switch from self-renewal to differentiation of ESCs. Of note, evidence deriving from miRNA-based reprogramming strategies underscores the role of the non-coding RNAs in the induction and maintenance of the stemness properties. In this review, we revised recent studies concerning the functions mediated by miRNAs in ESCs, with the aim of giving a comprehensive view of the highly dynamic miRNA-mediated tuning, essential to guarantee cell cycle progression, pluripotency maintenance and the proper commitment of ESCs.

## 1. Introduction

**Figure 1:**
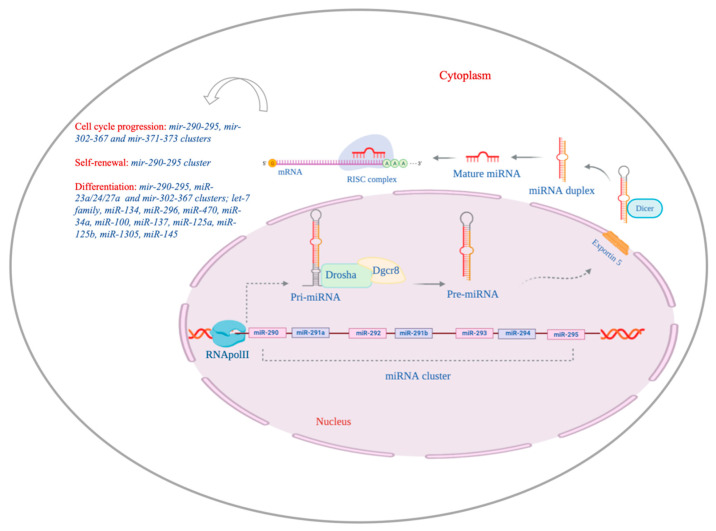
MicroRNA pathway in ESCs (embryonic stem cells). MiRNA genes can be organised in clusters as that of *mir-290*. Their biosynthesis starts in the nucleus when the RNA POL II generates a long primary transcript, with hairpin stem-loop structure, named pri-miRNA. The stem-loop cropping mediated by DROSHA/DGCR8 complex converts the pri-miRNA into a pre-miRNA, that reaches the cytoplasm through the EXPORTIN 5-mediated transport. Here, a processing of the pre-miRNA terminal loop by the endonuclease DICER generates a small miRNA duplex as intermediate. The mature miRNA strand is guided by RISC to bind the mRNA target, promoting its degradation/destabilization. MiRNAs orchestrate important functions as cell cycle regulation, self-renewal and differentiation of ESCs.

MicroRNAs (miRNAs) are small non-coding RNA molecules, approximately 23 nucleotides (nt) in length, able to regulate the expression of a large set of genes. They function by pairing with complementary sequences in mRNAs of protein-coding genes to direct their post-transcriptional repression [1]. In the last decade, miRNAs have emerged as functionally significant regulatory molecules in almost all cellular processes, from pluripotency to cellular aging [2,3]. As most RNA molecules, miRNAs undergo processing and then associate with proteins to regulate RNA degradation [4]. They are transcribed by RNA polymerase II (POL II) as a long (typically over 1 kb) primary transcript with hairpin stem-loop structure, called pri-miRNA, consisting of a stem of 33–35 bp, a terminal loop and single-stranded RNA segments at both the 5′ and 3′ sides (Figure 1) [5]. 

In the canonical pathway, the first step of the pri-miRNA processing occurs in the nucleus and involves a stem–loop cropping, mediated by the microprocessor complex containing RNase III-type enzyme DROSHA and the RNA-binding protein DGCR8 [6,7,8]. This precursor, called pre-miRNA (approximately 65 nt in length), is exported in the cytoplasm by EXPORTIN 5 and then cleaved near the terminal loop by the RNAse III type endonuclease DICER, generating a small miRNA duplex intermediate (approximately 22 nt in length) [9,10]. The small RNA duplex is loaded onto the Argonaute (AGO) protein to form the RNA-induced silencing complex (RISC) [11]. The AGO protein binds the duplex miRNA and incorporates the mature single strand miRNA, whereas the other strand is released [12]. The mature RISC complex leads to the repression of the target mRNA. Of interest, while the processing mediated by DGCR8 is restricted to miRNAs, the cytosolic DICER cleavage promotes the maturation of both miRNAs and short interfering RNAs (siRNAs) [10,13].

Often, miRNAs are organized in clusters and families. A “miRNA cluster” is encoded by the same locus in the genome and it can include distinct miRNAs belonging to different families and recognizing different mRNA targets (Figure 1). On the other hand, a “miRNA family” includes microRNAs sharing the similar “seed sequence”, the major determinant in target recognition and thus the same predicted targets [1]. MiRNA clusters or families may have functional relationships acting to coregulate or coordinately regulate biological processes. 

Stem cells are uncommitted cells able to differentiate, giving rise to somatic cell types. They exist in the embryo as well as in the adult organism, with a different developmental potential [14]. We can classify stem cells into: (1) totipotent stem cells (from zygote to 2-cell stage) able to differentiate in any kind of cells of both embryonic and extraembryonic tissues; (2) pluripotent embryonic stem cells found in early embryo, that can form any cell of the three germ layers (endoderm, ectoderm and mesoderm), but not the extraembryonic structures; (3) multipotent stem cells found in adults or in embryos with a more limited differentiation capacity, with their development limited to the cells that make up the organ system that they originated from. In pathological conditions such as cancer, other types of cells with stemness potential can populate the adult body and, therefore, they are named cancer stem cells. These cells, as a consequence of their stem-like potential, are characterized by an uncontrolled proliferation and can differentiate in heterogeneous cell lineages, generating new tumors during metastasis, as cancer initiating cells [15].

ESCs (embryonic stem cells) derive from the inner cell mass (ICM) of the mammalian blastocyst and show two outstanding properties: (i) self-renewal, described as the ability to endlessly propagate in culture in an undifferentiated state; (ii) pluripotency, described as the ability to generate all somatic and germline lineages of the embryo [16,17]. The pluripotency is a circuit managed by a complex network, in which transcription factors (TFs) and epigenetic regulators are the main actors [18,19,20,21,22,23,24]. ESCs cultured in vitro are a heterogenous population, composed of subpopulations that express fluctuating levels of the pluripotency TFs as well as a changeable propensity to differentiate [25]. This condition is defined as the metastable state of ESCs [26,27]. It is well known that an intricate network of miRNAs participates in the regulation of cell cycle, self-renewal and determination of the ESC identity (Figure 1) [28,29,30,31,32,33]. The pluripotency TFs OCT4, SOX2, NANOG and TCF3 promote the expression of miRNAs highly specific for ESCs and they even co-occupy, together with POLYCOMB proteins, the promoter regions of silent miRNA genes, later expressed during development. 

In 2006, Takahashi and Yamanaka demonstrated that somatic cells can be reprogrammed into induced pluripotent stem cells (iPSCs) by four TFs: OCT4, SOX2, KLF4, c-MYC [34]. These cells, like ESCs, can self-renew indefinitely and differentiate into all cell types, representing an attractive alternative to the use of ESCs. Starting from the Yamanaka discovery, great efforts have been made to improve reprogramming efficiency mainly by finding cocktails of factors that avoid genetic integration. Many reports have demonstrated that forced induction or repression of specific miRNAs can promote reprogramming to pluripotency or even induce the pluripotent state in somatic cells [35,36,37,38,39,40]. Thus, microRNAs contribute to stem cell maintenance and fate decisions as well as the establishment of pluripotency.

## 2. Embryonic Stem Cells and Pluripotency Transitions: An Overview

Pluripotency could be envisaged as a state represented by a developmental continuum of consecutive phases, named naïve, formative and primed [16]. Mouse ESCs (mESCs, embryonic day 3.5) and EpiSCs (epiblast stem cells, embryonic day 6.5) represent two different developmental stages, that mimic the naïve (pre-implantation) and the primed (post-implantation) pluripotency events [41,42].

**Figure 2:**
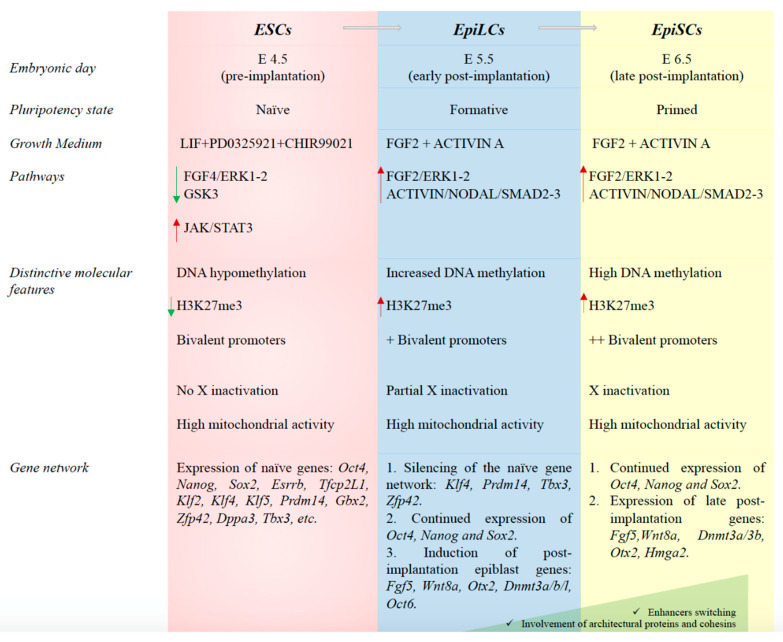
The different stages of pluripotency. Brief summary of the main molecular features defining the embryonic stem cells in the naïve, formative and primed states of pluripotency.

Naïve ESCs are assumed to be in an uncommitted state of pluripotency; they are able to generate all somatic lineages and contribute to chimera’s formation when injected in blastocysts [43]. Distinctive molecular features, such as DNA hypomethylation, low levels of the histone H3K27me3 modification, bivalent chromatin (marked by both H3K4me3 and H3K27me3), two active X chromosomes in female cell lines, and high mitochondrial content and glycolysis, characterize the cells in the naïve stage of pluripotency (Figure 2) [44,45,46]. A specific naïve pluripotency gene network (*Oct4, Nanog, Sox2*, *Essrb, Tfcp2L1, Klf2, Klf4, Klf5, Gbx2, Zfp42, Dppa3* and *Tbx3)* also characterizes ESCs in the ground state of pluripotency [17,43]. This network sustains self-renewal and is suppressed upon differentiation [16].

As uncommitted cells, the naïve ESCs must undergo maturation before taking a differentiation decision [16]. Formative pluripotency is the phase in which global changes, such as enhancer switching, DNA methylation changes and silencing of an individual X chromosome, occur to make the cells able to exit from the naïve state and switch to the primed state of pluripotency [47,48,49]. Although the pluripotency stages are in continuum in vivo, the formative pluripotency can be ideally considered as an intermediate state between the naïve and primed pluripotency. The undifferentiated state of ESCs is determined in vitro by pathways imposed by growth media composition [50]. The naïve state of pluripotency can be preserved in vitro by growing mESCs in a chemically defined media, named 2i, containing the leukemia inhibitory factor (LIF) and two small molecules PD0325921 and CHIR99021 [50]. 2i-treated ESCs are morphologically homogenous, show low levels of H3K27me3, have less bivalent domains and express optimal levels of the pluripotency markers compared to ESCs grown in presence of serum that, in contrast, are heterogenous in terms of morphology, transcriptome and epigenome [16,17,26,27]. Overall, 2i treatment has widespread effects on the transcriptome and epigenome of ESCs, while also impacting non coding RNA expression [26,27,51].

EpiSCs have been isolated from mouse post-implantation epiblasts and resemble cells of the late gastrula or primitive streak [52,53]. Although these cells are able to generate in vitro chimeras when grafted to post-implantation embryos and can differentiate into all the embryonic germ layers, they fail to contribute to in vivo chimeras after morula or blastocyst injection [16,54]. As opposed to naïve pluripotent stem cells, EpiSCs show increased amount of DNA methylation, undergo X inactivation and mainly exploit the glycolytic system for energy production. In addition to a less uniform expression of *Oct4*, *Nanog* and *Sox2*, the EpiSC gene network also includes the expression of *Dnmt3a/b, Fgf5, Pou3f1, Meis1, Otx2, Sox11* and *Gdf3* (Figure 2) [16,55,56]. The transition of the mESCs to formative pluripotent cells is mimicked in vitro by their differentiation into epiblast-like cells (EpiLCs) (around embryonic day 5.5) [48,56]. Indeed, ESCs grown in a chemically defined serum-free medium containing Fibroblast Growth Factor 2 (FGF2) and Activin A differentiate into EpiLCs [47,48,56]. This intermediate state separates pre- and post-implantation epiblasts and is reached 24–48 h after the cells have lost the ESC identity [47,56]. Although the EpiLC population is transcriptionally similar to post-implantation EpiSCs, it mimics the earlier post-implantation epiblast [47,52,56,57,58]. In EpiLCs, the naïve genes are switched off, the pluripotency factors *Nanog*, *Oct4* and *Sox2* continue to be expressed but at reduced levels compared to mESCs, and a subset of EpiSC genes (*Fgf5*, *Otx2* and *Oct6*) start to be expressed [47,56]. The expression of *Dnmt3l* also characterizes this intermediate state [56].

As for the murine counterpart, miRNAs fulfill crucial roles in both self-renewal and differentiation of human pluripotent stem cells (hPSCs). Interestingly, as reviewed below, the differences in developmental behavior between mouse and human PSCs lead to different biological effects of miRNAs in the two mammalian contexts. In this review, we take advantage from data deriving from the most recent studies to highlight how the fine tuning mediated by microRNAs in ESCs is essential to guarantee cell cycle progression and determination of cell fate. Importantly, the miRNA-mediated dynamics underlying the transition of ESCs from naïve to primed pluripotency state will also be addressed.

## 3. MicroRNA Machinery in ESCs: *Dgcr8* and *Dicer1* Knock-Out

In ESCs, miRNAs play different roles: they can act to maintain self-renewal or they can allow proper differentiation by suppressing pluripotency genes [59]. Significant evidence concerning miRNA regulation of stemness come from the detailed analysis of ESCs carrying deletions of the master genes involved in miRNA biogenesis and maturation. Several ESC lines in which the *Dgcr8* and *Dicer1* genes were knocked-out (*Dgcr8* and *Dicer1* KO ESCs) have been generated and characterized over the years. As expected, the detailed analysis of these cell models reveals the global loss of active miRNAs and their compromised maturation [60,61]. Of interest, these studies showed that miRNA-mediated regulation in ESCs was crucial mainly for the cell cycle progression rather than for pluripotency setting. Indeed, a proliferation defect was observed in both *Dgcr8* and *Dicer1* KO mESCs: although these cells were morphologically normal and express the pluripotency markers, they had an extended population doubling time, due to cell cycle arrest in G1 phase [60,61]. Detailed characterization of two independent *Dicer* KO mESC lines confirmed that *Dicer* loss impaired the exit from the pluripotency state as a consequence of cell cycle arrest in G1 and increased apoptosis [61,62]. Interestingly, *DICER1* seems to have a different role in hESCs (human embryonic stem cells), being required for their survival. Indeed, *DICER1* loss increased expression of pro-apoptotic genes and the apoptosis rate, leading to a failure of self-renewal without altering the cell cycle progression [63]. These differences between human and mouse ESCs could be due to their different developmental stage [42,52,56,58].

An additional distinctive characteristic of mESCs compared to hESCs concerns the differentiation defects described in both *Dgcr8* and *Dicer1* KO cells. In fact, the inability to exit from the pluripotency state of the *Dicer1*-deficient mESCs impairs their differentiation potential [62]. Likewise, the *Dgcr8* KO mESCs were not able to fully downregulate the pluripotency markers and abnormally expressed the differentiation markers [60].

Although the phenotypes of *Dgcr8* and *Dicer1* KO mESCs seem to be similar, some differences exist. First, the proliferation defect observed for *Dicer1* KO ESCs seems to be more profound than that described for *Dcgr8* KO cells. Second, the *Dicer1* KO cells stop their growth early during differentiation, while the *Dgcr8* KO cells grow and differentiate for an extended time [60,61,62]. As suggested by different studies, these differences could derive from additional roles for *Dicer* in ESC function, independent of miRNA biogenesis.

Finally, the importance of a “well-functioning” miRNA machinery in ESCs is further corroborated by studies conducted on *Ago* KO mESCs. AGO proteins, the main component of RISC complex, form ribonucleoprotein complexes involved in the transcriptional and post-transcriptional regulation of gene expression [64]. It has been demonstrated that the mESCs contain high levels of AGO proteins in the nucleus, where they assemble functional RISC complexes to induce post-transcriptional gene silencing [65]. In particular, the nuclear AGO proteins in ESCs are able to silence target mRNAs, binding sequences located in the coding region, in introns and in the 3′-UTR of their targets. *Ago2* KO ESCs can exit from the pluripotency state and, although they retain the ability to form the embryonic germ layers, they are unable to convert into extraembryonic endoderm cells [66]. Altogether, these observations indicate that the correct biogenesis and maturation of microRNAs is essential to guarantee the maintenance of the pluripotent state.

## 4. Naïve Pluripotency: The Relevant Role of the ESC-Specific Cell Cycle Regulating MiRNAs

The proper processing and expression of microRNAs has been described as essential for self-renewal as well as the establishment of the differentiation-competent state upon the exit from the naïve state (Table 1) [67,68,69].

ESCs have a shorter G1 phase than somatic cells, because they lack the restriction point (R-point), a cell cycle checkpoint initiated by external cues (i.e., nutrient starvation and cell–cell contacts) and mediated by Retinoblastoma (RB) protein phosphorylation [98]. Upon mESC differentiation, the R-point is newly established. Therefore, the regulation of the cell cycle length in ESCs is crucial for self-renewal persistence. Of special note, microRNAs are linked to their ability to repress inhibitors of the G1/S transition, promoting a fast shift from M to S phase and guaranteeing the rapid cell proliferation characteristic of stem cells [70]. Most notably, components of the *mir-290-295* cluster (*miR-290*, *miR-291a*, *miR-292*, *miR-291b*, *miR293*, *miR-294* and *miR295*), which are expressed at high levels in naïve mESCs, are downregulated upon differentiation [28,71,72,73,74]. Evolutionarily, this cluster derives from repeated duplications of the single microRNA *mir-290,* with its members acting at multiple levels of the Cyclin E-CDK2 pathway to promote efficient cell cycle progression [67,70]. Thus, the *mir-290–295* cluster post-transcriptionally regulates the expression of target mRNAs, such as *Cdkn1a* (or p21), *Rbl2* and *Lats2*, that have crucial roles in the G1/S transition [67,70]. Of note, these miRNAs, together with *miR-302*, have been defined as ESC-specific cell cycle-regulating miRNAs (ESCC miRNAs), because their introduction into *Dgcr8* KO ESCs rescued the proliferation defects [69]. ESCC miRNAs have redundant functions, share the same seed sequence (5′-AAGUGC-3′) and can influence cell cycle progression through both RB-dependent or -independent pathways [67,70,75]. *Mir-290-295* cluster also sustains the pluripotency of naïve ESCs, inducing stemness and enhancing glycolytic metabolism. Indeed, mESCs stably overexpressing this cluster maintain stemness properties such as pluripotency marker expression and colony formation ability, and unlike wild-type ESCs, do not undergo differentiation upon serum starvation [69]. Moreover, *mir-290/302* clusters enhance glycolysis in ESCs by targeting *Mbd2*, a methylated CpGs reader that negatively influences glycolysis and reprogramming [76]. Although the *mir-290* cluster is important for stemness maintenance, it can be considered dispensable for pluripotency setting of ESCs; in fact, the phenotypic analysis of *mir-290-295^−/−^* blastocysts revealed no morphological abnormalities or developmental defects [59,77]. Moreover, some studies have suggested that the *mir-290* cluster may regulate the early phases of differentiation. In 2008, it was demonstrated that this cluster guarantees the proper methylation state of pluripotency genes (i.e., *Oct4*) during differentiation through suppression of RBL2, a TF that negatively regulates the expression of the DNA methyltransferase genes *(Dnmt3)* [68]. More recently, it has been observed that *miR-294*, belonging to *mir-290-295* cluster, is sufficient to promote the differentiation of embryoid bodies into mesoderm and endoderm lineages [69]. However, the evidence is that ESCC miRNAs do not restore the differentiation defects described for *Dgcr8*-deficient cells, suggesting that multiple miRNA networks contribute to define ESC fate [70].

The *mir-290* cluster also contributes to the maintenance of ESC naïve state by controlling alternative splicing. *MiR-290* targets the negative regulator of the ESC-specific splicing code *Mbnl1/2* and, in turn, upregulates the expression of splicing factors in ESCs [78].

The maintenance of the bivalent state of developmental genes in the naïve ESCs is a further function of the *miR-290-295* family. As mentioned before, in undifferentiated ESCs, developmental genes are characterized by the coexistence of the histone modifications H3K4me3 (active state) and H3K27me3 (repressive state), a condition that defines their bivalent status [79]. This allows silencing of developmental genes in the undifferentiated state and, at the same time, primes them ready for activation. Towards this end, microRNAs belonging to the *miR-290-295* family are required for gene occupancy by the Polycomb Repressive Complex 2 (PRC2), a core component of many bivalent genes [80].

## 5. Selective Block of *let-7* MiRNAs Sustains the Naïve State of mESCs

The undifferentiated state of mESCs depends also on the suppression of differentiation-driving microRNAs. The selective block of miRNAs belonging to the *let-7* family is crucial for maintaining ESCs in their undifferentiated state. The *let-7* family is composed of 13 members encoded by 10 loci in the mouse genome, whose expression is tightly regulated; they are undetectable in undifferentiated ESCs and highly expressed in differentiated cells [32,72,99,100]. Although abundant levels of *pri-let-7-g* have been measured in undifferentiated ESCs, the maturation of this transcript is hampered by the RNA-binding proteins LIN28A and B. While already expressed in undifferentiated ESCs, LIN28A/B expression increases during the transition to primed state and decreases upon ESC differentiation [100,101,102]. *Lin28* paralogs work directly on *let-7* biogenesis at three different levels: In the nucleus, where LIN28B blocks the cleavage of *pri-let-7* by DROSHA; in the nucleoli, where LIN28B sequesters the *pri-let-7* transcript; and in the cytoplasm, where LIN28A blocks the DICER-induced processing of *pre-let-7*, inducing its oligouridylation and degradation [103,104,105]. In addition to the *let7*-dependent mechanisms, many results indicate that LIN28A also works through a *let-7*-independent mechanism by directly regulating many mRNAs. Depending on the mRNA target, LIN28 has been shown to both increase and decrease translation [100,106]. Thus, the mechanism of action of LIN28 seems to be context-dependent and based on the availability of its targets. Specifically, in the absence of *let-7,* or in presence of low *let-7* levels, LIN28A can bind its target mRNAs to regulate their translation [100,107,108]. This latter mechanism is particularly relevant in the first phases of ESC differentiation where *let-7* miRNAs are not expressed and thus LIN28 works by directly regulating mRNA translation [99,100]. Indeed, during the transition from ESCs to EpiLCs, the expression of the chromatin architectural protein HMGA2, required for the exit from the naïve pluripotent state, is tightly regulated by LIN28A in a *let-7*-independent manner [105,109].

Upon differentiation, embryonic stem cells switch off the expression of self-renewal genes and engage specific differentiation programs. One mechanism orchestrating the switch between self-renewal and differentiation depends on the antagonism between *let-7* and ESCC miRNAs. In fact, in the self-renewing state, the ESCC miRNAs indirectly promote the expression of the *Lin28* and *c-Myc* genes. Through this mechanism, LIN28 blocks accumulation of *let-7*, whereas c-MYC, together with the pluripotency genes N-MYC, OCT4, SOX2 and NANOG, induces the expression of the ESCC miRNAs. This positive feedback loop sustains the undifferentiated state of mESCs and inhibits their differentiation [32]. The ESCC miRNAs (*miR-294* and *miR-302*) also cooperate to allow the proper expression of self-renewal genes through the repression of the epithelial–mesenchymal transition and apoptotic pathways, two functions mediated by *let-7* [81]. Reduced expression of pluripotency genes at the initiation of differentiation results in depletion of *ESCC* miRNAs and LIN28. As a result, the block of *let-7* maturation is removed and it quickly accumulates. During this phase, *let-7* further suppress *Lin28* expression and downregulates *myc,* inhibiting the concomitant expression of ESCC miRNAs [32]. It also targets pluripotency genes to fix the differentiation state and inhibits the cell cycle progression, inducing the G1/S restriction point [75]. The results reviewed above demonstrate that the regulatory mechanisms underlying the state of ESCs are also based on dynamic regulation of microRNAs.

## 6. Naïve to Primed Pluripotency Transition: The Crucial Role of the *Mir-302* Cluster

MicroRNAs can be expressed in cell- and developmental-specific manners [110]. A recent meta-analysis evaluating data obtained from microRNA-seq, RNA-seq and metabolomics datasets from mouse and human ESCs revealed that 115 miRNAs show a differential expression profile during the naïve-to-primed transition [111]. These miRNAs act by repressing the developmental SONIC HEDGEHOG (SHH) pathway in the naïve state and regulating metabolic pathways such as oxidative phosphorylation, fatty acid metabolism and amino acid transport during the transition. As early as 2012, it was demonstrated that a peculiar miRNAs signature, including the differential expression of the *mir-290-295*, *mir-17-92* and *mir-302-367* clusters, distinguished the naïve and primed states of pluripotency [73]. As we already discussed, the *mir-290* cluster plays a dual role in stem cell differentiation; in fact, it is highly expressed in undifferentiated mESCs, but its expression is only moderately reduced during the transition from the naïve to the primed state of pluripotency [73]. This, together with the results discussed in the previous section of this review, suggests that this cluster orchestrates the transition from the naïve to the primed state [68,69]. A recent study revealed that the members of the *miR-290/302* family contributes to dismantling the naïve state by repressing pluripotency-related functions mediated by AKT and enhancing the activity of the differentiation-associated MEK pathway [74].

In contrast, the *mir-302* cluster is expressed at low levels in undifferentiated mESCs, with levels increasing during the ESCs to EpiLCs transition [73]. This dynamic expression profile suggests that the *mir-302* cluster has roles linked to primed pluripotency. The most important functions of this cluster have been identified in hESCs where the members of this cluster are highly expressed [84,112,113,114]. The hESC-specific miRNAs include members of the *mir-302-367* cluster (miR-302b *, *miR-302b*, miR-302c *, *miR-302c*, miR-302a *, *miR-302a*, *miR-302d*, and *miR-367*) and some components of the *mir-371-373* cluster [114,115]. The latter includes four members (*miR-371*, *miR-372*, *miR-373* * and *miR-373*) and it shares a common consensus sequence with the members of *mir-302-367* cluster [114]. Both these two hESC-specific miRNA clusters are conserved in the mouse genome: the murine homologous of *miR-302a* is *miR-302*, while the *mir-371-373* cluster represents the human homologue of the *mir-290-295* cluster [67,114].

As in mESCs, one of the primary functions of hESC-specific miRNA clusters concerns cell cycle regulation. The G1 cell cycle arrest observed in *DICER-* and *DROSHA*-deficient hESCs can be partially rescued by the introduction of *miR-195* and *miR-372*; these miRNAs negatively regulate the action of the tumor suppressors *WEE1* (negative regulator of G2/M transition) and *CDKNIA*, ensuring cell cycle progression [85]. Similarly, *miR-302* targets and post-transcriptionally represses the G1 phase regulator *CYCLIN D1,* as well as other negative regulators of G1 phase (*CDK4*, *RB*, *E2F1*, *P130*, *CDK2* and *CDK6*), to promote S phase entry [86]. In addition to regulation of the cell cycle, the components of the *miR-302-367* cluster have also been described as modulators of chromatin organization, vesicles transport, actin cytoskeleton and extracellular matrix remodelling, pluripotency and self-renewal of the hESCs [84]. The transcriptional regulation of *miR-302a* in hESCs is under the control of the pluripotency factors OCT4, SOX2 and NANOG, that bind the promoter region of this cluster, ensuring cell cycle progression [86]. Moreover, *miR-302* also sustains the pluripotent state by modulating the BMP pathway and repressing the neural differentiation [84].

## 7. The Exit from Naïve State and the Initiation of Differentiation: The Role of MicroRNAs

MiRNAs also play a central role in initiation of differentiation via their ability to suppress stemness-promoting pathways. In mESCs, three differentiation-associated miRNAs (*miR-134*, *miR-296* and *miR-470*) target the coding region of *Nanog*, *Oct4* and *Sox2* mRNAs, leading to the repression of these stemness factors and induction of differentiation [87,88]. *MiR-34a*, *miR-100*, and *miR-137* drive the differentiation of ESCs by modulating the expression of epigenetic regulators. The expression of these three miRNAs is induced upon the exit from the ESC undifferentiated state and correlates with the suppression of their targets, *Sirt1*, *Smarca5* and *Jarid1b*. The tight regulation of these epigenetic modifiers by miRNAs is required for ESCs to undertake a differentiation path. Indeed, the suppression of *miR-34a*, *miR-100*, and *miR-137* makes ESCs unable to leave the naïve state while, conversely, their overexpression induces aberrant activation of differentiation markers [89].

Among the mESC differentiation associated-miRNAs, *miR-27a* and *miR-24* are normally restrained by c-MYC and then released when self-renewal silencing must occur. During this stage, these miRNAs directly target the pluripotency-associated factors *Oct4*, *Foxo1* as well as the signal transducers of the self-renewal network *Smad2/3* (by *miR-27a*) and *Smad4* (by *miR-24*). This downregulation, in turn, represses c-MYC; therefore, *miR-27a* and *miR-24* expression levels are maintained steadily high, so that self-renewal is silenced, and the differentiation initiated [90,91].

**Figure 3:**
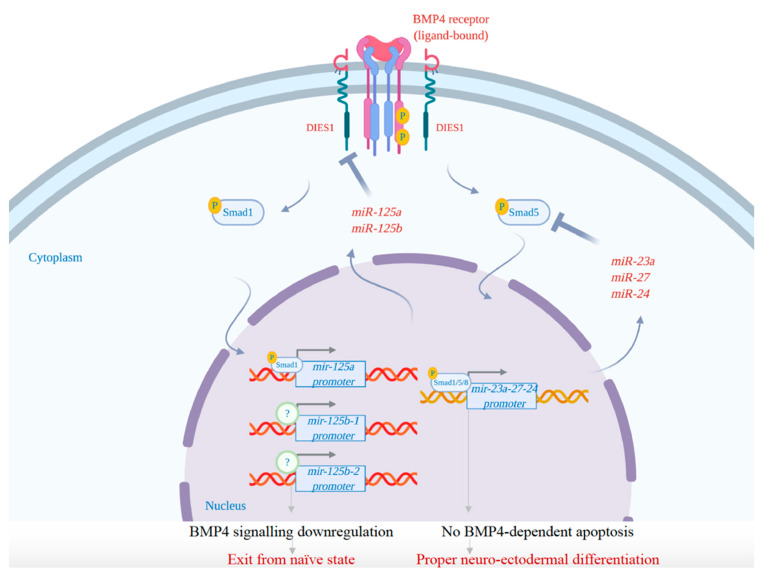
Feedback loop on BMP4 pathway mediated by *miR-125a/b* and *miR-23a-24-27a* during both the exit of mESCs from naïve state and their neuroectodermal differentiation. Briefly, *miR-125a* and *miR-125b* control the exit from the naïve state by targeting the BMP4 co-receptor Dies1, causing BMP4 pathway downregulation. In parallel, the *mir-23a-24-27a* cluster blocks the apoptotic response of mESCs to BMP4 signaling by targeting *Smad5* and allowing the proper differentiation of the neuroectodermal precursors.

Interestingly, data obtained by the suppression of members of *miR-23a-24-27a* cluster shed light on the crucial role of these miRNAs in the first phase of mESC differentiation. The expression of these miRNAs in ESCs is transcriptionally regulated by BMP4 through the recruitment of phospho-SMADs at the promoter of the gene encoding this miRNA cluster. These miRNAs are essential to protect the ESCs from apoptosis during differentiation. Indeed, they tightly control BMP4 signaling by targeting *Smad5* and generating an autoregulatory loop. This regulation is crucial to allow the proper differentiation of ESCs into neuroectodermal precursors. Although the suppression of these miRNAs does not affect self-renewal or pluripotency, it induces a significant increase of the cells undergoing apoptosis during the first days of differentiation. The expression of these miRNAs during the exit from the undifferentiated state is required to tightly regulate BMP4 signaling through the targeting of its effector SMAD5 [92]. BMP4 signaling represents a barrier to neuroectodermal differentiation: it can drive meso-endodermal differentiation (high signal) and can induce apoptosis of neuro-ectodermal precursors (low signal) [92,116]. Thus, the existence of a regulatory loop, involving SMAD5 and the *miR-23a* clusters, that acts to block the apoptotic response of differentiating ESCs to BMP4, is crucial to allow the establishment of neuroectodermal precursors. This fine mechanism acts in parallel with a similar feedback loop in which *miR-125a* and *miR-125b* target the BMP4 co-receptor DIES1 (now called VSIR), leading to the downregulation of BMP4 signaling during the exit from the naïve state [93,94,117]. Both mechanisms appear to be aimed at softening endogenous BMP4 signaling (Figure 3).

However, the two mechanisms are not merely redundant, as *miR-125a*-based loop controls the exit from the naïve state, whereas *miR-23a-24-27a* cluster functions soon after to counteract apoptosis of neuroectodermal precursors induced by BMP4, thus allowing progression of differentiation.

Similarly, the pluripotency/differentiation switch of hESCs is a tightly controlled process, in which specific miRNAs are downregulated in pluripotent cells. As for mESCs, specific differentiation-associated miRNAs have been reported to attenuate self-renewal and promote differentiation of hESCs (Table 2).

These miRNAs show a characteristic expression pattern, being almost undetectable in self-renewing cells, upregulated during the early differentiation, and downregulated during the later stages of differentiation [95,96]. For example, *miR-1305* has been proposed as a novel regulator of the cell cycle, as it is able to push G1/S transition and promote hESC differentiation through the post-transcriptional repression of *POLR3G*, an activator of the OCT4/NANOG pathway [95,97]. The suppression of the pluripotency program in hESCs often occurs through the downregulation of the key pluripotency factors and/or as a consequence of the repression of proteins involved in their pathways. For instance, Xu and colleagues reported a double negative feedback loop involving *OCT4*, *SOX2* and *KLF4* (OSK) and *miR-145* aimed at finely regulating the expression levels of these factors. In fact, *miR-145* suppresses self-renewal and induces differentiation by binding the 3′-UTR of OSK factors and repressing their expression [96]. A human specific *OCT4*-binding site on *miR-145* promoter also induced its repression, suggesting that, depending on the specific hESC stage, these two factors are able to influence each other [96].

## 8. MiRNAs and Long Non-Coding RNAs Orchestrate the Balance between Pluripotency and Differentiation in ESCs

Non-coding RNAs (ncRNAs) are a large class of RNA molecules that also include long non-coding RNAs (lncRNAs). The latter regulate gene expression by modulating transcription, RNA processing and translation [118]. As a consequence, they are involved in numerous biological processes, such as epigenetic regulation of chromatin remodeling, promoter specific gene-regulation, mRNA stability and X-chromosome inactivation [119]. LncRNAs also play an essential role in stem cell biology and can work by directly or indirectly interacting with miRNAs.

In hESCs, the intergenic lncRNA *LINC-ROR* functions as a key component of a feedback loop connecting miRNA networks and the core pluripotency TFs OCT4, SOX2, and NANOG. These stemness TFs induce the expression of *LINC-ROR* by transcriptional regulation [120]. Importantly, high levels of *LINC-ROR* in undifferentiated ESCs protect the TF core from *miR-145* targeting. Indeed, *LINC-ROR,* which shares miRNA-response elements with OCT4, SOX2, and NANOG, acts to sequester *miR-145* from these essential stemness factors. *LINC-ROR* expression levels also mimic that of the components of the TF core; in fact, its expression is restricted to undifferentiated ESCs and iPSCs and its level promptly decreases when the differentiation starts, preceding the TF downregulation [120,121]. This regulatory loop maintains a relative balance in self-renewing hESCs, limiting responses to subthreshold environmental stimuli, while at the same time promoting rapid and robust differentiation upon receipt of bona fide differentiation cues [120]. This mechanism describes the ability of lncRNAs to act as “microRNA sponge”, as competing endogenous RNAs (ceRNAs). To date, chemically synthesized competitive RNAs, having tandem binding sites for the target microRNAs, have been used as artificial microRNA inhibitors able to create a loss-of-function phenotype for an entire micro-RNA family in cell culture [121,122].

Another example of the interconnection between lncRNAs and miRNAs occurs during the decay of the naïve state and the setting of advanced states of pluripotency. The lncRNA *Ephemeron* (*Eprn*) fine tunes the dynamics of the cell state transition toward a state capacitated for lineage specification. This lncRNA is highly expressed in undifferentiated mESCs and its expression decreases during the transition into EpiSCs. *Eprn* downregulation causes decreased expression of the RNA binding protein LIN28A, resulting in the accumulation of *let-7g* miRNA and repression of the DNA methyltransferases *Dnmt3a/3b*, targets of *let-7g* in ESCs. As a final consequence, methylation of the *Nanog* proximal promoter is compromised and its expression is maintained, extending the transition latency from the naïve to formative pluripotency [82].

Recent evidence also highlighted the crucial role of the divergent lncRNAs in stem cell biology [123]. Divergent lncRNAs are transcribed in the opposite direction to nearby protein-coding genes and represent key players in the regulatory network governing ESC fate. The lncRNA *Trincr1* (TRIM71 interacting long noncoding RNA 1) has been described as regulator of the FGF/ERK signaling and self-renewal of ESCs. *Trincr1* KO causes a decrease of ESC self-renewal due to upregulation of phosphorylated ERK and of ERK pathway target genes [124]. *LncKdm2b* is another divergent lncRNA highly expressed in ESCs and early embryo. In agreement with this expression profile, the *LncKdm2b* knockout impairs ESC self-renewal and causes early embryonic lethality. *LncKdm2b* works by activating the transcription of the TF *Zbtb3* that, in turn, promotes *Nanog* expression to potentiate ESC self-renewal [125].

Several studies revealed that divergent lncRNAs can also regulate ESC differentiation. *Evx1as* is a lncRNA able to regulate the transcription of nearby genes. In particular, it is required to promote the expression of the *Evx1* gene during the differentiation of mESCs. This mechanism is based on the binding of *Evx1as* to regulatory sites on the *Evx1/Evx1as* locus that promotes chromatin looping and facilitates Mediator binding to the promoter. The expression of *Evx1as* is required to allow the proper meso-endodermal differentiation of ESCs [126]. The lncRNA *DIGIT* is conserved in mouse and human and its expression is induced during endoderm differentiation of both mESCs and hESCs. The absence of DIGIT in ESCs leads to a deficiency in definitive endoderm differentiation also due to the failure of *Gsc* gene activation. Indeed, DIGIT is not only divergently transcribed from the gene encoding Goosecoid (GSC) but it also promotes *Gsc* expression by acting *in trans* [127].

The identification of new lncRNAs and microRNAs as well as the discovery of new lncRNA-miRNA axes is “a crescendo” changing our concept of transcriptome.

## 9. MiRNAs and Epigenetic Regulation in ESC Self-Renewal and Differentiation

Increasing evidence indicates that the expression of miRNAs as well as their function is closely related to the complex epigenetic regulation occurring in PSCs and that orchestrates the balance between self-renewal and differentiation. Both transcription and biogenesis of microRNAs undergo epigenetic control by DNA methylation and histone modifications. The study of Glaich and colleagues demonstrated that the DNA methylation state of the genes encoding for microRNAs influences the maturation of the relative pri-miRNAs [128]. Indeed, the methyl-CpG binding protein 2 (MECP2) binds to methylated miRNA loci, slowing POL II-mediated elongation and recruiting DGCR8 to the nascent pri-miRNA. Then, the microprocessor complex enhances the primary miRNA processing. On the other hand, in the absence of DNA methylation, POL II-mediated elongation is fast, and DROSHA is unable to bind to the nascent pri-miRNAs. Moreover, the authors also demonstrated that biogenesis of the miRNAs encoded by highly methylated DNA regions is more perturbed upon changes in methylation than that of miRNAs encoded by unmethylated DNA [128].

DNA methylation can also have the classical repressive effect on miRNA transcription in ESCs. Indeed, the expression of the miRNAs encoded by the *Dlk1-Dio3* imprinted gene cluster in mESCs requires the activity of PRC2 to prevent the recruitment of DNMT3 and the subsequent de novo DNA methylation. This mechanism allows the proper expression of this miRNA cluster in undifferentiated ESCs [129]. Of note, many papers have pointed out the crucial role of miRNAs in controlling the expression and/or the activity of epigenetic regulators in PSCs. The members of both PRC1 and PRC2 are finely regulated by miRNAs in mESCs. CBX7, the specific PRC1 “reader” of the H3K27me3 mark, is highly expressed in undifferentiated ESCs and downregulated during differentiation. The tight control of its expression is necessary to allow the exit from the naïve state. Indeed, ectopic expression of *Cbx7* inhibits differentiation, whereas its knockdown induces differentiation and de-represses the lineage-specific markers. *MiR-125* and *miR-181* families are induced during ESC differentiation and directly control the downregulation of *Cbx7* to allow proper differentiation [130]. Two components of PRC2 complex are found to be regulated by specific miRNAs in mESCs. The mRNA of *embryonic ectoderm development (Eed)* gene, the PRC2 “reader” of the H3K27me3 mark, is directly targeted by *miR-323-3p*. The binding of *miR-323-3p* to *Eed* mRNA results in reduced EED protein abundance and decreased H3K27me3 levels indicating that *miR-323-3p* can regulate the function of PRC2 by modulating *Eed* expression [131]. *Enhancer of Zeste 2 (Ezh2) gene*, the enzymatic component of the PRC2 complex, is regulated by *miR-214* during differentiation of mESCs induced by retinoic acid. Indeed, ESC differentiation is accompanied by increased expression of *miR-214* and, conversely, reduced EZH2 protein levels, indicating that *miR-214* could reduce EZH2 and de-repress transcription of developmental regulators to allow differentiation of ESCs [132].

As mentioned in the previous sections of this review, the *mir-290* cluster is required for the binding of EZH2 and Suppressor of Zeste 12 (SUZ12) at many bivalent promoters, and therefore, for the maintenance of the bivalent state of ESCs [80]. The control of the PRC activity could not be attributed to changes in the expression levels of PRC1 and PRC2 but rather, miR-290 members regulate the targeting of PRC1 and PRC2 to appropriate loci in ESCs to maintain their undifferentiated state. A different study confirmed this hypothesis. Indeed, Kanellopoulou and colleagues demonstrated that the *Hox* genes, which are associated with ESC differentiation, are regulated at transcriptional level by *mir-290* cluster. Interestingly, this control is due to a reduced localization of PRC2 at specific loci [133]. Polycomb Group Proteins (PcGs) maintain mESCs in a pluripotent state by silencing the *Hox* genes and other “bivalent” differentiation genes primed for transcription. *MiR-291* directly represses the methyltransferase *Ash1l*, which can activate the *Hox* genes by evicting POLYCOMB during differentiation, confirming that the PRC targeting is influenced by *miR-290* miRNAs in ESCs [133].

As mentioned before, the *mir-290* cluster contributes to epigenetic control in mESCs also by indirectly regulating DNMT3. Indeed, the miRNAs of the 290 cluster target the transcriptional repressor of *Dnmt3a/b*, *Rbl2*, thus allowing the de novo DNA methylation required during ESC differentiation [68].

The post-transcriptional and epigenetic control by miRNAs also functions to regulate ESC state. Indeed, a dual-repressive molecular circuit was described in mESCs. This circuit, involving PRC and ESCC microRNAs, regulates the expression of the endocytosis-associated genes (EAGs). Some EAGs are bound and repressed by the PRC and, at the same time, they are further subjected to post-transcriptional regulation by *miR-294*, indicating the existence of a “dual mechanism” of gene repression required to maintain the pluripotent state of ESCs [134].

## 10. Conclusions

The in vitro differentiation of mouse ESCs can be used to mimic embryonic development, thus allowing the study of fundamental mechanisms of gene expression regulation. During the earliest phases of differentiation, ESCs undergo pluripotency transitions, passing through the naïve, formative and primed states, that reproduce the embryonic stages from pre-implantation to early post-implantation development. The role of key TFs in these transitions is well documented. Of note, many reports indicate that miRNAs carry out a fundamental role in regulating ESC fate decisions [83]. The correct biogenesis and maturation of microRNAs is essential to guarantee the normal continuum of the pluripotency phases during mammalian development [60,62,66]. Therefore, the role of the non-coding small RNAs cannot be considered as secondary to that of transcription factors. Recent reports have outlined the impact of miRNAs on shaping the transcriptional profiles of pluripotent stem cells also at a single cell level. Single cell transcriptome sequencing of *Dgcr8* KO ESCs upon introduction of single miRNAs as *miR-294* and *let-7c,* highlighted the opposite effects of these two miRNAs on the co-expression of cell cycle phase genes and cellular heterogeneity of these cells. Indeed, *miR-294* decreases the heterogeneity between cells and suppresses the phasing of cell cycle genes, whereas *let-7c* increases transcriptional heterogeneity, and promotes the co-expression of G2/M cell cycle phase genes [83]. Another recent study revealed that ESCs exhibit intrinsic heterogeneity in the absence of external gradients by forming interconverting cell states with distinct gene expression programs and miRNA activities [135]. MiRNAs contribute to increased variation of target genes and cell states. Indeed, the loss of miRNAs delayed the transitions across cell states suggesting that miRNAs play also a central role in organizing fluctuations across gene networks to coordinate and promote state transitions.

In this review, we provided a comprehensive depiction of the miRNA networks acting in ESCs, with the purpose of demonstrating that microRNAs significantly contribute to changes in gene expression occurring during the naïve-to-primed transition, as well as the early stages of differentiation. Interestingly, the regulation of exit from the naïve state also rests on the establishment of feedback loops, where fine-tuning of gene expression by miRNAs allows ESCs to properly respond to extrinsic signals that may have multiple effects on differentiation. We can imagine that many other miRNAs can be engaged in such complex regulatory mechanisms.

The continued identification and characterization of miRNA-based networks regulating stem cell fate will expand opportunities to control the pluripotent stem cell differentiation for therapeutic purposes.

## Figures and Tables

**Table 1:** Main microRNAs acting in ESCs and their relative functions.

MiRNA Cluster/Families	ES-Specific Functions	References
*mir-290-295* cluster (*miR-290, miR-291a, miR-292, miR-291b, miR-293, miR-294, miR-295*)	- cell cycle progression through post-transcriptional repression of inhibitors of the CYCLIN E-CDK2 pathway;	[67,68,69,70,71,72,73,74,75,76,77,78,79,80]
	- induction of stemness properties;	
	- glycolysis enhancement by post-transcriptional repression of *Mdb2*;	
	- up-regulation of splicing factors through post-transcriptional repression of *Mbn1/2*;	
	- maintenance of bivalent state of the developmental genes;	
	- induction of early differentiation and methylation of pluripotency genes through post-transcriptional repression of *Rbl2*;	
	- naïve pluripotency dismantling by repression of AKT-mediated functions;	
	- enhancement of differentiation associated pathways (MEK).	
*let-7* family (*let-7a, let-7b, let-7c, let-7d, let-7e, let-7f, let-7g, let-7i, miR-98, miR-202*)	- induction of ESC differentiation and suppression of LIN28;	[32,75,81,82,83]
	- downregulation of *ESCC miRNAs*;	
	- inhibition of cell cycle progression;	
	- induction of epithelial-mesenchymal transition;	
	- apoptosis.	
*mir-302-367* cluster (miR-302b *, miR-302b, miR-302c *, miR-302c, miR-302a *, miR-302a, miR-302d, miR-367)	- induction of primed pluripotency;	[30,74,76,81,84,85,86]
	- induction of S phase entry by post-transcriptional repression of *CYCLIND1* and others negative regulators of the G1 phase;	
	- regulation of chromatin organization, vesicles transport, actin cytoskeleton, extracellular matrix constituents, pluripotency and self-renewal;	
	- reprogramming of mouse and human somatic cells in iPSCs in absence of transcription factors.	
*mir-371-373* cluster (miR-371, miR-372, miR-373 *, miR-373)	- cell cycle regulation by post-transcriptional repression of *WEE1* and *CDKNIA.*	[85]
*miR-134, miR-296, miR-470*	- targeting of pluripotency factors *Nanog*, *Oct4* and *Sox2* leading to mRNA repression and mESC differentiation.	[87,88]
*miR-34a, miR-100, miR-137*	- transcriptional repression of *Sirt1*, *Smarca5* and *Jarid1b* with consequent ESC differentiation.	[89]
*miR-23a/24/27a cluster*	- targeting of *Oct4*, *Foxo1*, *Smad2/3* (by *miR-27a*) and *Smad4* (by *miR-24*) for self-renewal silencing and ESC differentiation.	[90,91,92]
	- regulation of BMP4 signaling by *Smad5* targeting to allow the establishment of neuroectodermal precursors and avoid BMP4-induced apoptosis.	
*miR-125a, miR-125b*	- downregulation of BMP4 pathway by targeting the BMP4 co-receptor *Dies1* to guarantee the proper differentiation of mESCs.	[93,94]
*miR-1305, miR-145*	- post-transcriptional repression of *POLR3G*, with consequent downregulation of the key pluripotency factors.	[95,96,97]
	- self-renewal suppression and induction of differentiation by post-transcriptional repression of *OCT4*, *SOX2* and *KLF4*.	

**Table 2:** MicroRNAs expression and roles in hESCs (human embryonic stem cells) vs. mESCs (mouse ESCs).

MicroRNA Clusters/Families	Expression in hESCs or mESCs	Biological Function
*mir-290-295* cluster	Highly expressed in mESCs	Regulation of naïve pluripotency, cell cycle progression and early phases of differentiation.
*mir-371-373* cluster	Highly expressed in hESCs	Regulation of cell cycle and stemness maintenance.
*mir-302-367 cluster*	Present in both hESCs and mESCs, but highly expressed in hESCs	Regulation of pluripotency, self-renewal and reprogramming.
*let-7 family*	Higly expressed in both differentiating hESCs and mESCs	Regulation of naïve to primed pluripotency transition.
*miR-134, miR-296, miR-470, miR-34a, miR-100, miR-137, miR-27a, miR-24, miR-125a, miR-125b*	Expressed in differentiated mESCs	Differentiation-associated miRNAs.
*miR-372, miR-195, miR-1305, miR-145*	Expressed in differentiated hESCs	Differentiation-associated miRNAs.

## Abbreviations

ESCs	embryonic stem cells
EpiSCs	epiblast stem cells
miRNAs	microRNAs
nt	nucleotide
POL II	RNA Polymerase II
AGO	argonaute
RISC	RNA-induced silencing complex
siRNAs	short interfering RNAs
ICM	inner cell mass
TFs	transcription factors
iPSCs	induced pluripotent stem cells
LIF	leukemia inhibitory factor
EpiLCs	epiblast-like cells
hESCs	human embryonic stem cells
hPSCs	human pluripotent stem cells
KO	knock-out
R-point	restriction point
RB	retinoblastoma
PRC	polycomb repressive complex
SHH	sonic Hedgehog
ncRNAs	non-coding RNAs
lncRNAs	long non-coding RNAs
Eprn	ephemeron
PcGs	polycomb group proteins
EAGs	endocytosis-associated genes
